# Influence of shape, size and magnetostatic interactions on the hyperthermia properties of permalloy nanostructures

**DOI:** 10.1038/s41598-019-43197-4

**Published:** 2019-04-29

**Authors:** Riccardo Ferrero, Alessandra Manzin, Gabriele Barrera, Federica Celegato, Marco Coïsson, Paola Tiberto

**Affiliations:** 1 Istituto Nazionale di Ricerca Metrologica (INRIM), Torino, Italy; 2 Politecnico di Torino, Torino, Italy

**Keywords:** Biomedical engineering, Magnetic properties and materials

## Abstract

We present a detailed study of permalloy (Ni_80_Fe_20_) nanostructures with variable shape (disk, cylinder and sphere) for magnetic hyperthermia application, exploiting hysteresis losses for heat release. The study is performed modifying nanostructure aspect ratio and size (up to some hundreds of nanometres), to find the optimal conditions for the maximization of specific heating capabilities. The parameters are also tuned to guarantee negligible magnetic remanence and fulfilment of biophysical limits on applied field amplitude and frequency product, to avoid aggregation phenomena and intolerable resistive heating, respectively. The attention is first focused on disk-shaped nanostructures, with a comparison between micromagnetic simulations and experimental results, obtained on nanodisks still attached on the lithography substrate (2D array form) as well as dispersed in ethanol solution (free-standing). This analysis enables us to investigate the role of magnetostatic interactions between nanodisks and to individuate an optimal concentration for the maximization of heating capabilities. Finally, we study magnetization reversal process and hysteresis properties of nanocylinders (diameter between 150 nm and 600 nm, thickness from 30 nm up to 150 nm) and nanospheres (size between 100 nm and 300 nm), to give instructions on the best combination of geometrical parameters for the design of novel hyperthermia mediators.

## Introduction

Magnetic hyperthermia is a promising tumour therapy that exploits magnetic nanostructures, typically superparamagnetic iron oxide nanoparticles (SPIONs), and alternating magnetic fields, to increase the temperature of diseased tissues^1–5^. Many studies^6–12^ have demonstrated that a rise in temperature *T* of tumour cells can improve chemotherapy or radiotherapy efficacy due to cellular alteration (*T* = 42–45 °C) or can directly lead to cell death (*T* > 46 °C), even in treatments lasting only minutes (thermo-ablation^5^).

In magnetic hyperthermia, magnetic nanostructures are injected into the tumour and then excited by an ac magnetic field, with a given frequency that ranges from 50 kHz to 1.2 MHz. This leads to a localized release of heat and to a consequent increment of the tissue temperature. Three independent mechanisms result in thermal energy, namely Néel relaxation, Brownian relaxation and hysteresis^13–15^. Their contribution strongly depends on the size, shape, magnetocrystalline anisotropy and degree of aggregation of the nanostructures. Also eddy currents can concur to thermal energy production, but in a negligible way for the frequency range typically considered^15^.

One of the main limitations of magnetic hyperthermia is the medium heating efficiency of the currently used magnetic nanostructures, which have to be injected in large quantity to obtain therapeutic effects, at the cost of potential toxicity^4^. Coated SPIONs are considered promising candidates for hyperthermia applications, because of good biocompatibility and weak tendency to agglomeration^16,17^. However, their heating efficiency is limited, since thermal energy comes from size-dependent Néel and Brownian relaxation, with negligible hysteresis loss contribution. In a tumour tissue, Brownian relaxation is practically inhibited due to nanoparticle immobilization, apart from a small oscillation allowed by tissue elasticity^18^. A similar behaviour is also found in the cellular environment, where Brownian losses are not present, due to nanoparticle attachment to the cellular membrane or confinement within endosomes^19^. Studies on iron-oxide nanoparticles (5–22 nm size) showed that, for a diameter of ∼10 nm, Néel losses reach their maximum and then their relative heating contribution starts reducing as diameter increases. The emerging single-domain ferromagnetic behaviour leads to an increase in hysteresis losses, which overcome Néel losses for nanoparticle dimensions higher than 15–20 nm^20,21^.

Both single- and multi-domain ferromagnetic nanostructures have recently attracted strong interest, because of the potential improvement of heating efficiency due to hysteresis losses. Different strategies have been adopted to tune hysteresis properties with the aim of increasing the relative heating contribution. A possible way is to use materials with high saturation magnetization and/or high uniaxial magneto-crystalline anisotropy, like cobalt or cobalt-zinc ferrites^19,22–26^. Another strategy consists in modifying nanostructure geometry, introducing shape anisotropies; promising results were obtained with magnetite nanorods^27^, maghemite, magnetite or cobalt ferrite nanocubes^28–30^ and octahedral magnetite nanoparticles^31^.

Nanostructure geometry can strongly affect magnetization reversal process and thus remanence magnetic state, which should be characterized by negligible moment to reduce magnetostatic interactions and agglomeration effects. To this aim, special attention has been given to nanodisks, nanorings and nanotubes, which exhibit magnetic vortex configuration at remanence and thus very small remanence moment^32–35^. They are able to preserve some of the advantages of SPIONs (reduced aggregation and good colloidal stability), enabling to obtain large hysteresis losses too.

Hysteresis losses can be augmented by increasing the amplitude *H*_*a*_ of the applied field up to saturation conditions and its frequency *f*. However, when a patient is exposed to an ac magnetic field, parameters *H*_*a*_ and *f* should be controlled to avoid the undesirable induction of eddy currents in the body with the occurrence of hot-spots, causing possible discomfort and tissue damage. To limit these effects, restrictions are imposed on the product *H*_*a*_ *f*; an upper value of 4.85 · 10^8^ Am^−1^s^−1^, known as the Atkinson-Brezovich limit^36^, was first assumed as an acceptable threshold to avoid intolerable resistive heating. A less rigid criterion, *H*_*a*_ *f* ≤ 5 · 10^9^ Am^−1^s^−1^, was successively proposed^37^. Good tolerability was documented during ones of the first clinical trials on magnetic hyperthermia, performed on patients with different tumour types, which were exposed to a field of 100 kHz, with amplitude variable between 2.5 kA/m and 18 kA/m^38,39^. In silico and *in vivo* analyses demonstrated the possibility of overcoming the threshold of 5 · 10^9^ Am^−1^s^−1^, maintaining eddy current effects below safe and tolerable limits^40^. As an example, breast and pancreatic tumours in mice were successfully treated with SPIONs exposed to magnetic fields with *H*_*a*_ = 15.4 kA/m and *f = *435 kHz^41^.

Finally, it is fundamental to estimate the minimum field necessary to achieve a good heating efficiency, factor to be considered in the choice of the working frequency. If saturation conditions can’t be reached due to biophysical limits, large hysteresis losses could be still obtained by properly tuning the field and amplitude of irreversible jumps via nanostructure shape and size modification.

Here, we present a modelling analysis of permalloy (Ni_80_Fe_20_) nanostructures with variable shape (disk, cylinder and sphere), for possible application in magnetic hyperthermia. A parametric study is performed by modifying nanostructure aspect ratio and size (up to some hundreds of nanometres), to find the optimal conditions for the maximization of the specific heating capabilities with tolerable fields. Hysteresis losses, which are the main heating contribution for the considered nanostructures, are calculated by integrating the Landau-Lifshitz-Gilbert (LLG) equation^42^, with the inclusion of thermal noise effects.

The attention is first focused on disk-shaped nanostructures, for which we present a detailed comparison between experimental and numerical results. The studied nanodisks, with diameters ranging from 270 nm to 680 nm, are prepared by means of a nanolithography technique based on the self-assembling of polystyrene nanospheres^43,44^. Hysteresis loop measurements are performed on samples in two spatial configurations, namely in 2D array form (nanodisks still attached on the substrate) and dispersed in an ethanol solution (free-standing nanodisks). Both experimental conditions are reproduced in the modelling analysis, to provide an interpretation of the obtained results, with specific attention to the role of magnetostatic interactions.

In a second step, we study magnetization reversal process and hysteresis properties of nanocylinders (diameter between 150 nm and 600 nm, thickness from 30 nm up to 150 nm) and nanospheres (size between 100 nm and 300 nm). We analyse hysteresis losses, remanent magnetization state and quasi-saturation conditions, as a function of size and shape. The aim is to find the optimal geometrical properties, which guarantee energy release maximization, negligible magnetic remanence and fulfilment of the biophysical constraint on *H*_*a*_ *f*.

## Materials and Methods

### Sample fabrication and dimensional characterization

Disk-shaped permalloy nanostructures are prepared by means of a bottom-up nanolithography technique based on the self-assembling of polystyrene nanospheres^43,44^. The nanofabrication procedure consists in sputtering a continuous thin film of permalloy on a Si substrate, coated with a layer of optical resist. Successively, a monolayer of polystyrene nanospheres is deposited on the permalloy film and properly reduced in diameter by plasma etching in Ar^+^. The polystyrene nanospheres are then used as a hard mask for sputter etching with Ar^+^ ions the exposed areas of permalloy. Subsequently, the hard mask is removed by sonication in deionised water, resulting in a 2D array of permalloy nanodisks on the resist layer surface. Finally, the nanodisks are detached from the substrate, by chemically dissolving the underlying resist.

**Figure 1:**
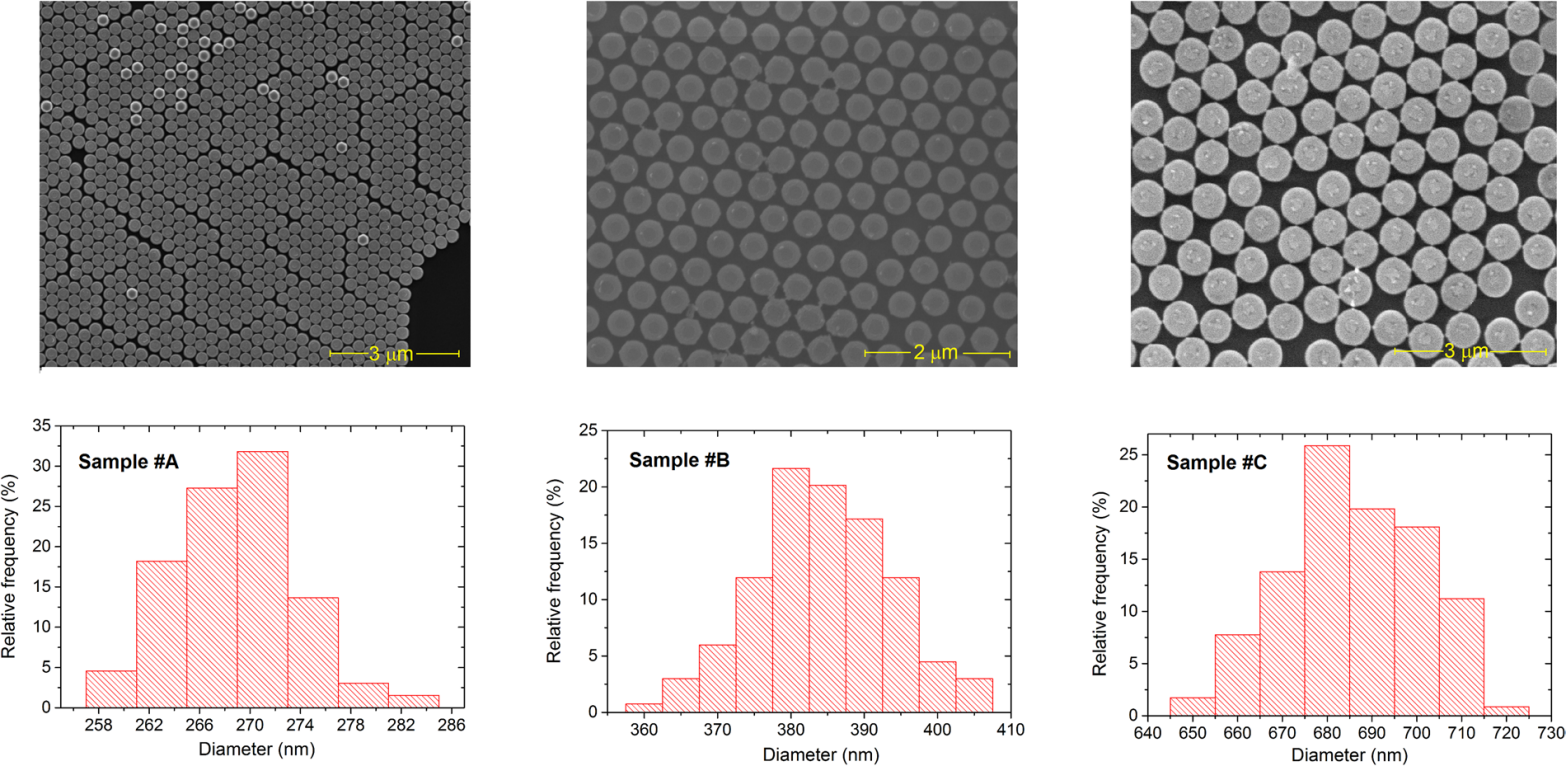
Top: Scanning electron microscopy (SEM) images of permalloy nanodisk arrays prepared by self-assembling of polystyrene nanospheres with initial diameter of 300 nm (sample #A), 500 nm (sample #B) and 800 nm (sample #C). Bottom: graphs of the statistical distribution of nanodisk diameters. All samples have an average thickness of 30 nm.

We produce samples with three sizes, which depend on the initial diameter of the polystyrene nanospheres used for the lithographic mask (nominal values of 300 nm, 500 nm and 800 nm) and on the duration of plasma etching process. The dimensional characterization is performed in an intermediate stage of the fabrication process, when the nanodisks are still attached on the substrate, forming a 2D array with quasi-hexagonal lattice. The diameter distribution is derived by analysing a set of Scanning Electron Microscopy (SEM) images of different areas of the 2D arrays (top of Fig. 1) by means of *ImageJ* software^45^. Image processing enables us to obtain the statistical distributions of the nanodisk diameters (bottom of Fig. 1), corresponding to mean diameters of 270 nm (sample #A), 380 nm (sample #B) and 680 nm (sample #C), and relative standard deviations of 5 nm, 9 nm and 14 nm. Via SEM image analysis we also determine the centre-to-centre distance among disks, which results to be in the order of 300 nm (sample #A), 480 nm (sample #B) and 780 nm (sample #C). For all the samples, the thickness of the nanodisks is 30 ± 1.5 nm, as derived from a priori calibration of the sputter deposition of permalloy film.

### Hysteresis loop measurements

First, we perform magnetic measurements of disk-shaped permalloy nanostructures in the 2D array form, a stage where their spatial distribution can be easily reproduced in the modelling analysis. Static hysteresis loops are obtained in a temperature range from 10 K to 300 K, in order to extrapolate the temperature dependence of saturation magnetization, remanent magnetization and coercive field. This characterization is done by means of an ultra-sensitive alternating-gradient field magnetometer (AGFM) equipped with a liquid-He continuous flow cryostat.

Second, we measure the room-temperature hysteresis loop of free-standing nanodisks in ethanol suspension, by using a Vibrating-Sample Magnetometer (VSM). The ferrofluid is placed within a sample holder suitable for VSM.

Microstructure and, consequently, magnetic properties of the sputtered permalloy films and of the derived nanodisks strongly depend on the deposition conditions (e.g. working gas pressure and substrate temperature)^46^. In particular, high deposition pressures can induce porosity or void structure leading to a variation in the film density, which results lower than the bulk one^47^. As a consequence, also the saturation magnetization of the thin films and of the derived nanodisks is decreased with respect to the nominal bulk value (in the order of 800 kA/m for permalloy). For this reason, we previously perform an experimental characterization of the saturation magnetization of a permalloy continuous film, having thickness comparable to the one of the layers used for the nanodisk production and obtained with the same deposition parameters. The saturation magnetization measured at 300 K results to be ∼570 kA/m: this value is set as a reference for both hysteresis loop measurements and micromagnetic modelling.

### Micromagnetic modelling

The micromagnetic modelling of permalloy nanostructures is performed by numerically integrating the LLG equation

$$\frac{\partial {\bf{M}}}{\partial t}=-\,\frac{\gamma }{({\rm{1}}+{\alpha }^{{\rm{2}}})}{\bf{M}}\times [{{\bf{H}}}_{eff}+\frac{\alpha }{{M}_{S}}({\bf{M}}\times {{\bf{H}}}_{eff})]$$

where **M** is the magnetization vector with amplitude equal to the saturation magnetization *M*_*S*_, *γ* is the absolute value of the gyromagnetic ratio and *α* is the damping coefficient, fixed to 0.02. The effective field **H**_*eff*_ is the sum of applied external field, magnetostatic field, exchange field and magnetocrystalline anisotropy field, which is assumed to be negligible for permalloy. Moreover, it includes the thermal field **H**_*th*_, here calculated by means of the Langevin approach, namely

$${{\bf{H}}}_{th}={\boldsymbol{\eta }}({\bf{r}},t)\sqrt{\frac{2\alpha {k}_{B}T}{\gamma {\mu }_{0}{M}_{S}{\rm{\Delta }}{s}^{3}{\rm{\Delta }}t}}$$

where *k*_*B*_ is the Boltzmann constant, *T* is the absolute temperature, *μ*_0_ is the vacuum magnetic permeability and **η** is a stochastic vector whose components are Gaussian random numbers, uncorrelated in space and time, with zero mean value and dispersion 1^48,49^. Moreover, Δ*s* is the average length of the spatial discretization, in the order of the exchange length (∼5 nm), and Δ*t* is the time discretization step.

The update of the LLG equation is performed by means of a norm-conserving time integration scheme based on the Cayley transform and on the Heun method^50,51^, under the assumption of uniform magnetization vector in each discretization element.

To speed up the computation, we calculate the effective field terms with different parallelized solvers, depending on the sample spatial arrangement (2D array form or 3D random distribution) and on the nanostructure type (2D-approximable or 3D). For the nanodisks arranged in 2D arrays or randomly distributed in a 3D domain, we calculate the magnetostatic field via a Fast Multipole method, suitable for the treatment of large-scale samples composed of different objects or unit cells^42,52,53^. The exchange field is calculated with a finite difference technique able to treat non-structured meshes and thus appropriate for describing the curved edges of nanodisks^54^. This technique is particularly convenient for nanodisks randomly oriented in the space, since it avoids the introduction of fictitious shape anisotropy effects, which arise when structured meshes are used in presence of geometrically anisotropic objects not aligned with grid axes.

For 3D nanostructures, the magnetostatic field is calculated via an FFT approach based on the discrete convolution theorem. In this case, the samples are discretised with a structured mesh and a standard finite difference method is used for the determination of the exchange field.

## Results

### Experimental and modelling analysis of nanodisks in 2D array form

We focus here on disk-shaped nanostructures with vortex based magnetization switching^55^, analysing the influence of diameter on hysteresis losses for samples in 2D array form.

**Figure 2:**
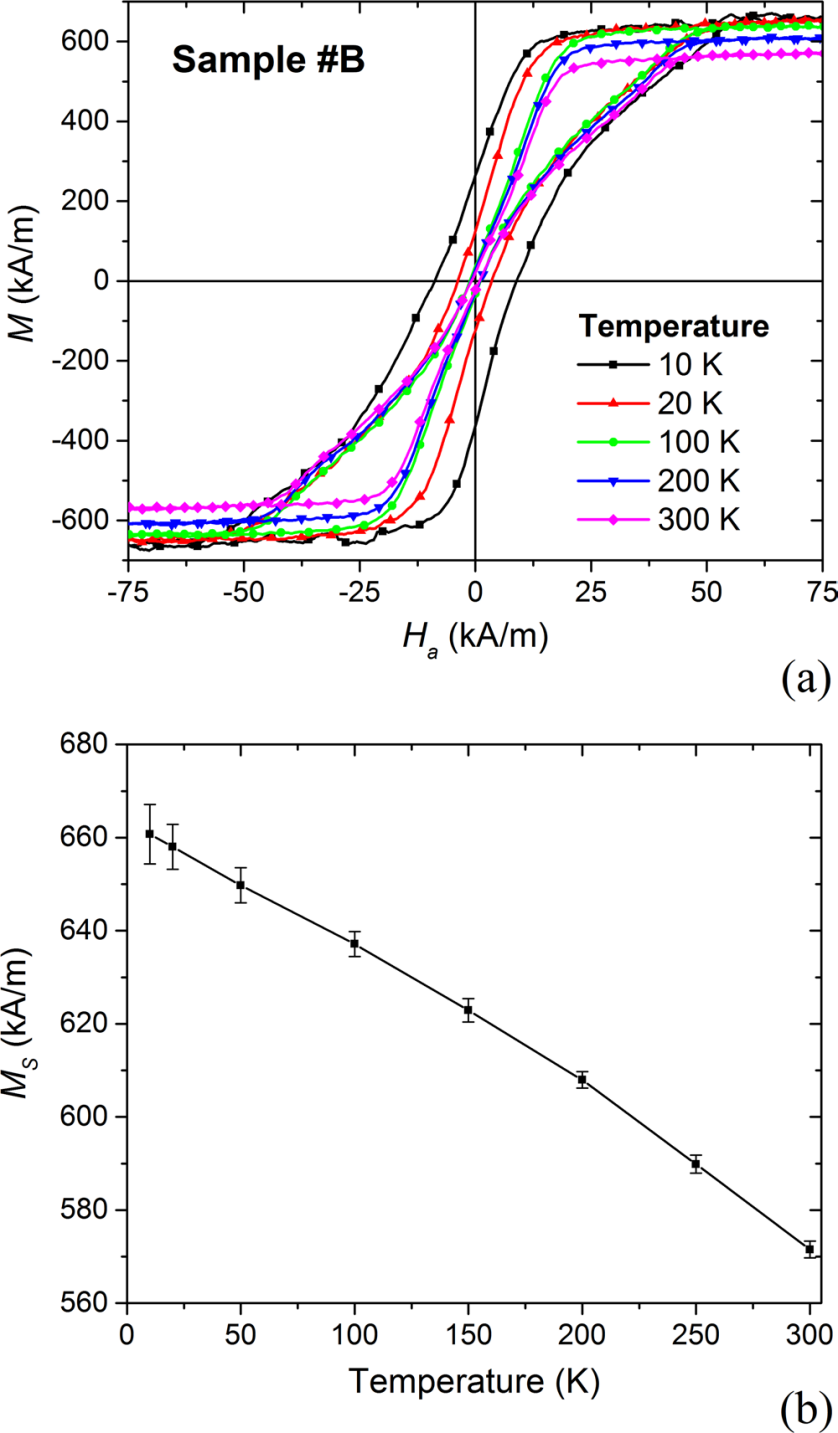
(**a**) Hysteresis loops of sample #B (mean disk diameter of 380 nm) in 2D array form, measured at different temperatures from 10 K to 300 K. (**b**) Saturation magnetization versus temperature for sample #B, from experimental characterization.

Figure 2a reports a set of static hysteresis loops measured at different temperatures (from 10 K to 300 K) for sample #B. At very low temperatures, we find high coercive field and remanent magnetization values, strongly decreasing between 10 K and 20 K. Above 20 K the two quantities keep decreasing, but at a much slower rate. As shown in Fig. 2b, also the saturation magnetization *M*_*S*_ reduces with temperature; at 300 K *M*_*S*_ is decreased of about 15% compared to the measurement at 10 K (570 kA/m versus 670 kA/m). The value of *M*_*S*_ at low temperature, which results less than the nominal one, is well justified by the reduction in sputtered permalloy density (6.19 g/cm^3^ versus 8.72 g/cm^3^).

**Figure 3:**
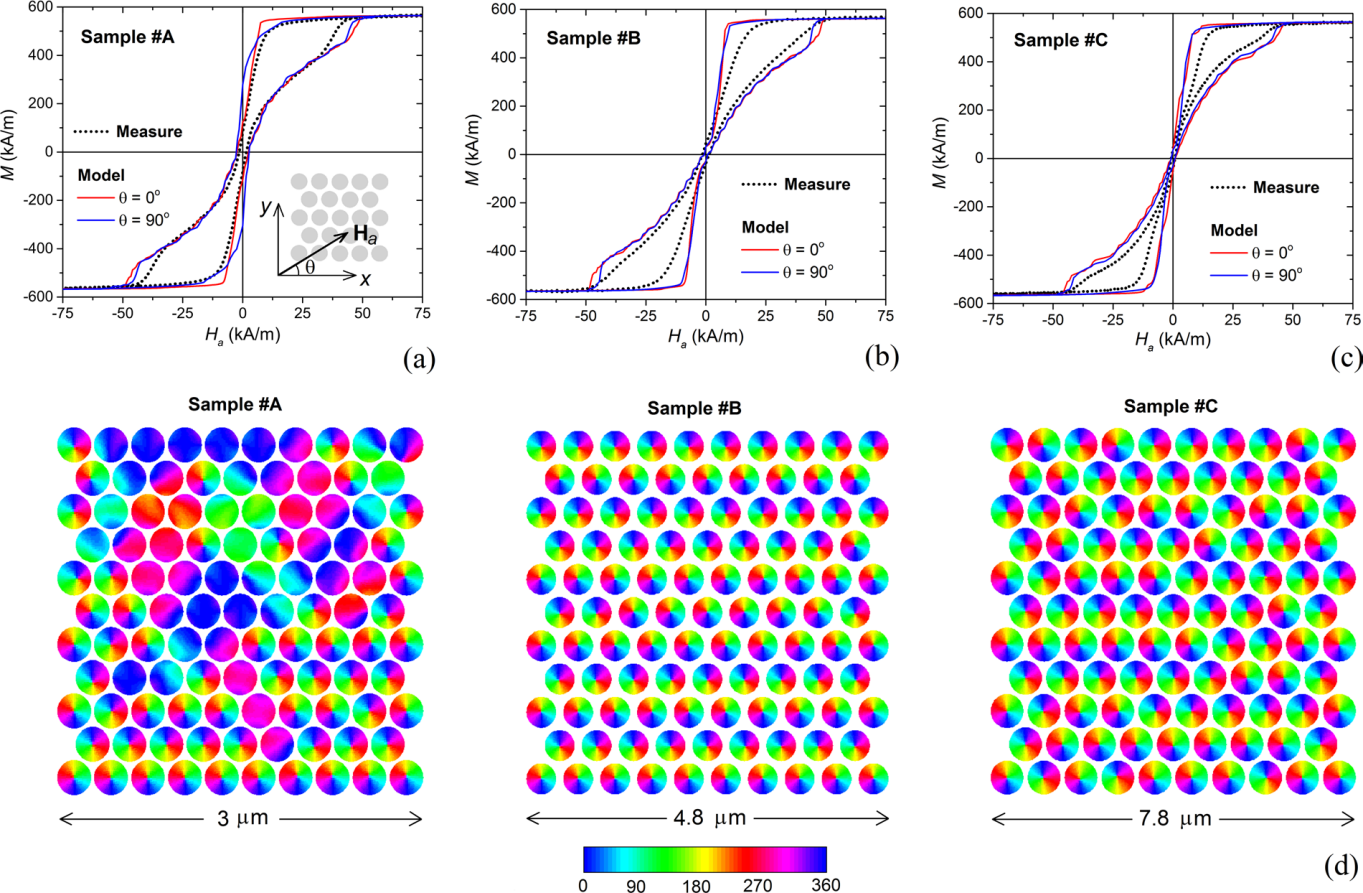
Top: Comparison between measured and calculated hysteresis loops of the disk-shaped nanostructures in 2D array form at temperature of 300 K, for nanodisks with mean diameter equal to (**a**) 270 nm (sample #A), (**b**) 380 nm (sample #B) and (**c**) 680 nm (sample #C). Simulation results are obtained by applying the external field both along *x*- (θ = 0°) and *y*-axis (θ = 90°) directions [inset in (**a**)]. Bottom (**d**): calculated magnetization configurations at remanence for external field applied along *x*-axis (the colour bar represents the angle, in degrees, between magnetization vector and *x*-axis). The results are reported in the following order: on the left: sample #A; in the centre: sample #B; on the right: sample #C.

After this preliminary study, we compare the static hysteresis loops measured at 300 K to the calculated ones for all the three samples (top of Fig. 3). In the micromagnetic simulations, which include thermal noise effects, the nanodisk arrays are described as ordered patterns with hexagonal packing, considering the mean diameter and the mean centre-to-centre distance extracted from SEM images. The numerical results are obtained with the following material properties: saturation magnetization of 570 kA/m (from experimental characterization); exchange constant of 13 pJ/m; zero magnetocrystalline anisotropy.

As demonstrated by Fig. 3, modelling results are in good agreement with experimental ones for all samples, with a reliable prediction of the magnetization reversal process. Small discrepancies in vortex nucleation and expulsion fields can be explained by patterning imperfections in the fabricated samples, which are characterized by edge roughness and variations in nanodisk diameter and reciprocal distance, as shown by the SEM images in Fig. 1. This stochastic distribution of defects can facilitate the transition between states, due to the creation of artificial pinning and depinning sites for vortexes. As a consequence, vortex nucleation and expulsion result anticipated in the measured loops. Moreover, magnetization transitions are more gradual, due to the non-synchronous nucleation and expulsion of vortexes, caused by the random distribution of pinning/depinning sites. Additionally, patterning imperfections lead to an isotropic behaviour, strongly mitigating the influence of hexagonal lattice on shape anisotropy.

The role of microstructure lattice can be evinced by modelling results, which are obtained under the assumption of ordered patterns. Modelled sample #A, for which the minimum distance δ between nanodisk boundaries is fixed to 30 nm, shows a weak geometrical six-fold anisotropy with easy and hard axes alternating every 30°, as demonstrated by the comparison of the hysteresis loops calculated along the hard and easy axes. These correspond to angular field orientations θ (inset in Fig. 3a) equal to 0° (*x*-axis) and 90° (*y*-axis), respectively, and to geometrically equivalent directions, shifted in multiples of 60°. Due to the magnetostatic interactions between nanodisks, we find non-negligible values for the remanent magnetization and the coercive field, higher when θ = 90°. This effect can be seen also in the corresponding measured loop.

The collective switching behaviour is confirmed by the equilibrium magnetization state calculated at remanence for θ = 0° (Fig. 3d, left). In particular, not all the nanodisks are in the vortex state, due to non-synchronous vortex nucleation, which results to be spread over a large field interval around zero. Moreover, in some nanodisks vortex nucleation is inhibited and magnetization reversal takes place via the formation of C-state, due to the mutual interaction with neighbouring nanodisks.

Modelled samples #B and #C (δ = 100 nm) have a more isotropic behaviour, with remanent magnetization and coercive field close to zero and limited differences between the two considered field orientations. Their behaviour tends towards the one of the single disk, with synchronous magnetization switching. This is illustrated by the equilibrium magnetization states calculated at remanence when θ = 0°, shown in Fig. 3d (centre, for sample #B, and right, for sample #C). At zero field all the nanodisks are in vortex state, with more centred vortex for sample #B, which is characterized by a higher ratio of centre-to-centre distance to diameter (reduced intensity of magnetostatic interactions).

For both measurements and simulations, hysteresis losses reduce with the nanodisk diameter increase. The measured specific energy losses are about 20 kJ/m^3^ for sample #A, 16 kJ/m^3^ for sample #B and 10 kJ/m^3^ for sample #C. The calculated energy losses are moderately higher, being approximately 27 kJ/m^3^ for sample #A, 25 kJ/m^3^ for sample #B and 20 kJ/m^3^ for sample #C (average of data obtained with θ = 0° and θ = 90°).

### Experimental and modelling analysis of free-standing nanodisks

**Figure 4:**
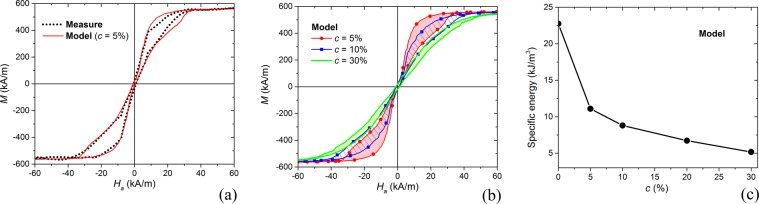
(**a**) Comparison of room-temperature measured hysteresis loop of ∼680 nm diameter nanodisks dispersed in ethanol solution with the loop calculated at 300 K for a volume concentration of 5%. (**b**) Comparison of simulated hysteresis loops for different nanodisk concentrations ranging from 5% to 30%. (**c**) Specific energy losses calculated as a function of nanodisk concentration. All the numerical results are obtained by fixing the nanodisk diameter to 680 nm.

Now, we characterize free-standing nanodisks dispersed in ethanol, focusing on sample #C (mean diameter of 680 nm). During hysteresis loop measurement, the nanodisks do not deposit on the sample holder bottom and remain suspended in the solution with a random spatial distribution, thanks to the dynamic nature of the VSM technique. The resulting room-temperature loop (Fig. 4a) presents a more gradual reversal than the loop for the corresponding sample in the 2D array form (Fig. 3c). In this case, it is not possible to identify vortex nucleation/expulsion transitions, since magnetization switching is not synchronous (vortexes are generated and expelled at different applied fields). This is a consequence of the magnetostatic interactions between nanodisks as well as of the variation in the angular orientation of nanodisks with respect to the applied field direction. The local misalignment between the nanodisk plane and the field affects the loop shape, since the reduction in the in-plane field component requires the application of fields with larger amplitudes to enable both vortex nucleation and expulsion.

The experimental behaviour can be well interpreted by means of micromagnetic simulations. These are performed by randomly distributing the nanodisks in a 3D space with different volume concentrations (from 5% up to 30%) and setting the temperature at 300 K. Very diluted systems are also simulated, considering a single nanodisk. The volume concentration *c* is defined as the ratio *V*_*nanodisks*_/*V*_*system*_, where *V*_*nanodisks*_ is the volume of a nanodisk multiplied by the number of nanodisks and *V*_*system*_ is the minimum volume of the 3D domain containing all the nanodisks.

A good agreement with measurements is obtained when *c* is ∼5%, as demonstrated by the comparison in Fig. 4a. The measured specific energy losses are ∼8 kJ/m^3^, while the calculated ones are ∼11 kJ/m^3^. This energy amount can be obtained by applying a sufficiently high field, in the order of 40 kA/m, which guarantees to reach quasi-saturation state. Regarding biophysical constraints^36,37^, the above field amplitude is acceptable if we assume as a limit *H*_*a*_ *f* ≤ 5 · 10^9^ Am^−1^s^−1^ and as a maximum frequency 125 kHz. In this case, a specific loss power (SLP) around 150–200 W/g can be obtained. The estimated value is in line with the results in the review article by Angelakeris^40^, considering that the reported data were obtained on nanoparticles made of different materials, with a product *H*_*a*_ *f* that exceeds more than twice the limit of 5 · 10^9^ Am^−1^s^−1^. Very high values, up to ∼5 kW/g, were found for iron oxide nanodisks, under the exposure to an external field with amplitude of 47.8 kA/m and frequency of 488 kHz^33^. However, a non-negligible remanence magnetization value, around 100 kA/m, is observed.

As shown in Fig. 4b, the increase in *c* leads to an enhancement of the magnetostatic interactions, resulting in a wider spread of vortex nucleation/expulsion fields. This causes an increment of the saturation field, thus limiting the maximum acceptable frequency to avoid resistive heating. Moreover, it has a detrimental effect on the specific energy losses, which reduce from ∼23 kJ/m^3^ for extremely diluted systems (negligible magnetostatic interactions) down to ∼5 kJ/m^3^ for concentrations around 30% (Fig. 4c). Thus, high volume concentrations lead to a strong reduction in the heating efficiency, with negative repercussions for hyperthermia applications. The obtained result is in agreement with the conclusions of Haase and Nowak, which investigated the effects of magnetostatic interactions for increasing concentrations of magnetic nanoparticles^56^. Similar findings were reported by Martinez-Boubeta *et al*., when studying the influence of concentration on the heating properties of iron oxide nanocubes in aqueous solutions^29^. Analogously, Guibert *et al*. observed that the SLP of iron oxide nanoparticles strongly decreases when moving from well-dispersed systems to large and dense aggregates^57^. Moreover, in the study of multi-core iron oxide nanoparticles, Blanco-Andujar *et al*. found that the core-to-core magnetostatic interaction can adversely affect magnetic heating properties^58^.

**Figure 5:**
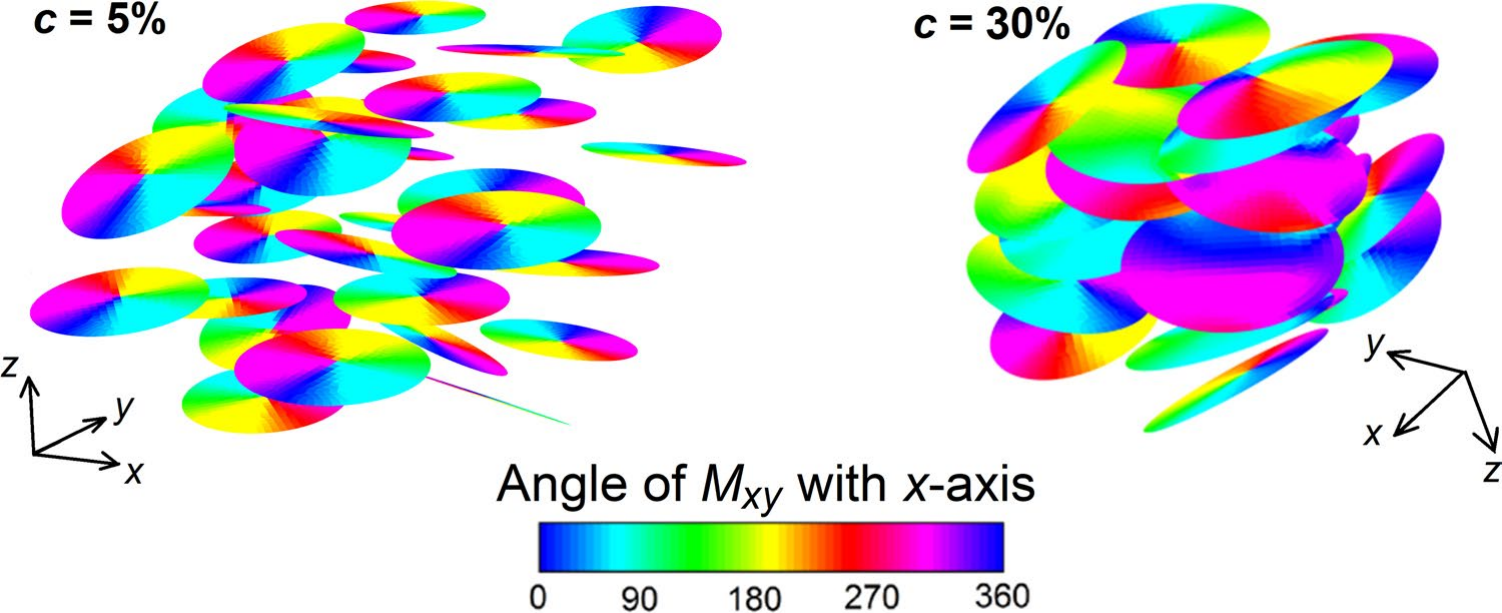
Calculated magnetization configurations at remanence for external field applied along *x*-axis (the colour bar represents the angle, in degrees, between magnetization component in the *xy*-plane and *x*-axis). The reported results refer to the case of 680 nm diameter nanodisks randomly distributed in a 3D domain with volume concentrations of 5% (left) and 30% (right). The temperature is set at 300 K.

The influence of nanodisk concentrations on magnetization reversal processes is finally investigated, by analysing the evolution of magnetization configuration at successive equilibrium points along the hysteresis loop descending branch. The obtained results, reported in Fig. S1 of Supplementary Information for volume concentrations of 5% and 30%, demonstrate that the nucleation and expulsion of vortexes are not synchronous, due to both magnetostatic interactions and local misalignment with the applied field. This effect results amplified for the highest concentration, leading to a remanent state where not all the nanodisks are in the vortex configuration, as can be seen in Fig. 5.

### Modelling analysis of the role of nanostructure size and shape

Via micromagnetic modelling, we here determine the hysteresis losses of permalloy nanostructures with different shape (disk, cylinder or sphere) and variable size in the sub-micrometer range. The aim is to find optimal geometrical properties for hyperthermia applications, reaching a compromise between the maximization of the produced heat and the fulfilment of biophysical constraint on the applied field. The simulations are performed considering the nominal value of magnetization saturation for permalloy (800 kA/m) and including the effects of thermal noise, fixing the temperature to 300 K.

First, we calculate the hysteresis loops of nanocylinders with diameters *d* ranging from 150 nm to 600 nm and variable thickness *t* starting from 30 nm. The field is applied in the plane perpendicular to the nanocylinder axis. For all the considered geometries, the nucleation of an out-of-plane vortex (with core perpendicular to the applied field axis) is expected^55,59^.

**Figure 6:**
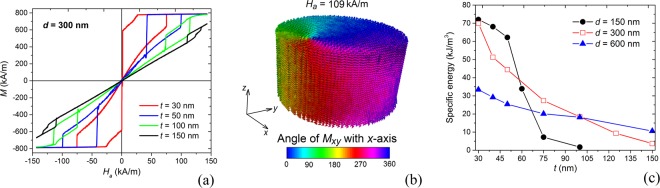
(**a**) Comparison of hysteresis loops calculated as a function of thickness *t* ranging from 30 nm to 150 nm, for nanocylinders with diameter *d* equal to 300 nm. The external field is applied orthogonally to the cylinder axis and the temperature is set at 300 K. (**b**) Case *d* = 300 nm and *t* = 150 nm: magnetization configuration showing the out-of-plane vortex that forms immediately after the first irreversible jump. The colour bar represents the angle, in degrees, between magnetization component in the *xy*-plane and *x*-axis (applied field direction). (**c**) Specific energy losses calculated as a function of nanocylinder thickness for diameter *d* equal to 150 nm, 300 nm and 600 nm.

As shown in Fig. 6a, which reports the results obtained for *d* = 300 nm, the loop shape is strongly influenced by *t*. Its increase leads to a rise in out-of-plane vortex nucleation and expulsion fields, due to the reduction in shape anisotropy, typical of disk geometry.

For high values of *t*, e.g. *t* = 150 nm, the quasi-saturation state with in-plane magnetization is no more energetically favoured. The out-of-plane vortex configuration is stable for a very large field range, leading to a dominant reversible behaviour and thus to a small loop area. The magnetization reversal starts with the formation of a third-order buckle state^55^, which evolves into a vortex lying in the plane perpendicular to the applied field and with core moment pointing in the field direction. At the first irreversible jump, occurring at ∼110 kA/m, the in-plane vortex transforms into a vortex with out-of-plane core and magnetization rotating in the plane orthogonal to the nanocylinder axis. Along the reversible part of the loop, the out-of-plane vortex moves orthogonally to the applied field up to the opposite side, where it is expelled around −117 kA/m, evolving into a third-order buckle state. The final irreversible jump, occurring at about −125 kA/m, corresponds to the shift of the buckle state towards the applied field direction.

At remanence, the magnetization rotates symmetrically around the nanocylinder axis, resulting in a negligible moment. The evolution of magnetization configuration at successive equilibrium points along the hysteresis loop descending branch is illustrated in Fig. S2 of Supplementary Information. Figure 6b shows the magnetic state, with out-of-plane vortex, which forms after the first irreversible jump.

When increasing *t*, we observe a reduction in the hysteresis loop area and a rise in out-of-plane vortex nucleation and expulsion fields also for nanocylinder diameters equal to 150 nm and 600 nm. The calculated loops are reported in Fig. S3 of Supplementary Information.

The behaviour of specific energy losses as a function of geometrical parameters *d* and *t* is depicted in Fig. 6c. The graph puts in evidence how the increase in *d* and *t* diminishes hyperthermia efficiency; the highest energy value, around 72 kJ/m^3^, is obtained for *d* = 150 nm and *t* = 30 nm. For the specific case of *d* = 150 nm, we observe a slight decrease in the hysteresis losses when varying *t* from 30 nm to 50 nm, followed by an abrupt reduction between 50 nm and 75 nm, due to the weakening of shape anisotropy. Very low hysteresis losses are found for diameters higher than 100 nm, similarly to what happens for *d* = 300 nm and *t* = 150 nm.

For the nanocylinders with *d* equal to 300 nm and 600 nm the specific energy losses decrease more gradually with *t*, due to the higher aspect ratio *d*/*t*. However, for *t* = 30 nm, the doubling of *d* from 300 nm to 600 nm causes the halving of produced heat.

Hysteresis losses can be maximized by applying sufficiently large fields, which enable out-of-plane vortex expulsion and, thus, the reaching of quasi-saturation state. Moreover, loop areas can be amplified by reducing vortex nucleation fields and increasing vortex expulsion fields as much as possible. At the same time, the nanostructures should be properly designed to avoid too high vortex expulsion fields, whose application at the frequencies typical for hyperthermia could lead to intolerable resistive heating.

**Figure 7:**
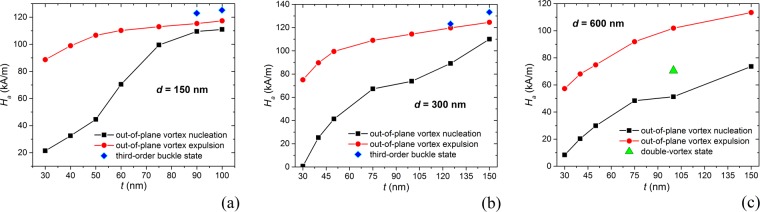
Out-of-plane vortex nucleation and expulsion fields versus thickness *t*, extracted from the hysteresis loops of the nanocylinders with diameter equal to (**a**) 150 nm, (**b**) 300 nm and (**c**) 600 nm, calculated at 300 K. The reported values are obtained by averaging the data obtained along the descending and ascending hysteresis loop branches. In (**a**) and (**b**) the blue rhombic markers reported for high values of *t* correspond to the field of the last irreversible jump, which is not characterised by the out-of-plane vortex expulsion, but to the transition to a third-order buckle state. In (**c**) the green triangle marker at *t* = 100 nm corresponds to the field at which the transition to double-vortex configuration takes place.

To further investigate the suitability of permalloy nanocyliders for magnetic hyperthermia, in Fig. 7 we report out-of-plane vortex nucleation and expulsion fields versus thickness for the diameters of 150 nm (a), 300 nm (b) and 600 nm (c). For the 150 nm diameter cylinders with *t* ranging from 30 nm to 100 nm, the nucleation field rises from ∼20 kA/m up to ∼110 kA/m, rapidly increasing between 50 nm and 75 nm, in correspondence with the abrupt reduction in energy losses (Fig. 6c). The out-of-plane vortex expulsion field slightly increases, varying from ∼90 kA/m to ∼120 kA/m. For *t* around 90–100 nm, the last irreversible jump does not correspond to the expulsion of the out-of-plane vortex, but to the transition to a third-order buckle state, as described for *d* = 300 nm and *t* = 150 nm. For the 600 nm diameter cylinders, the out-of-plane vortex expulsion and nucleation fields increase nearly at the same rate and, consequently, hysteresis losses reduce more gradually with *t*. When *t* is around 100 nm, between quasi-saturation and out-of-plane vortex nucleation there is an intermediate state, characterized by a double-vortex configuration, illustrated in Fig. S4 of Supplementary Information.

Considering the biophysical constraint *H*_*a*_ *f* ≤ 5 · 10^9^ Am^−1^s^−1^, the high expulsion fields reported in Fig. 7 strongly impact on the largest acceptable value for *f*. This should be in the order of 50 KHz, resulting in a maximum allowable field of 100 kA/m. To satisfy the above requirement, we should opt for nanocylinders with limited thickness, e.g., when *d* = 150 nm (300 nm), *t* should be lower than 40 nm (50 nm). It follows that disk-shaped nanostructures with a diameter of few hundred nanometres (less than 300 nm) could be the optimal solution in terms of both energy release and fulfilment of the applied field constraint. In this case, an SLP value higher than 400 W/g can be obtained.

**Figure 8:**
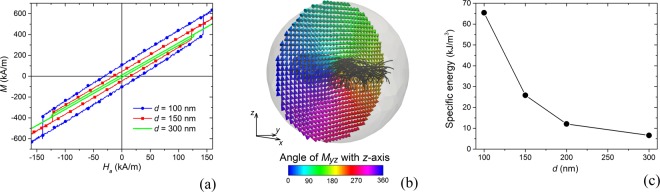
(**a**) Comparison of the calculated hysteresis loops for nanospheres with diameter *d* ranging from 100 nm to 300 nm. The simulations are performed by fixing the temperature to 300 K. (**b**) Remanence magnetization configuration for the nanosphere with diameter equal to 150 nm. The external field is applied along *x*-axis. Magnetization vector distribution is reported for the central *yz*-plane. The colour bar represents the angle, in degrees, between magnetization component in the *yz*-plane and *z*-axis; the streamlines describe the vortex core. (**c**) Specific energy losses calculated as a function of nanosphere diameter *d*.

Finally, we investigate the suitability of permalloy nanospheres for hyperthermia applications, focusing on diameters *d* ranging from 100 nm to 300 nm (see the hysteresis loops in Fig. 8a). For these nanostructures, vortex nucleation and thus saturation condition can be reached at very large fields, higher than 150 kA/m. For lower fields, the reversal mechanism is almost entirely a reversible process. As illustrated for *d* = 150 nm in Fig. S5 of Supplementary Information, the magnetization prevalently rotates around the applied field axis, with the exception of the centred vortex core, which results aligned with the external field^60^. Also at remanence (Fig. 8b for *d* = 150 nm), the vortex core contributes with a non-negligible magnetic moment, maintaining its orientation up to the irreversible jump. Here, the magnetization in the vortex core switches, following the new direction of the applied field. The field inversion gives rise to a hysteresis loop symmetric around zero, whose size depends on the nanosphere diameter. In particular, remanent magnetization and irreversible jump field reduce by increasing *d* (Fig. 8a). This strongly impacts on the specific energy losses, which diminish from ∼65 kJ/m^3^ to ∼7 kJ/m^3^ when increasing the diameter from 100 nm to 300 nm.

Moreover, it is interesting to note that the change in the nanosphere size has a weak effect on the diameter of the vortex core (*d*_*core*_), which is about 40 nm. This explains the decreasing behaviour of specific energy losses versus *d*, which can be interpolated by a function $f(d)=a{d}_{core}^{2}/{d}^{2}$, where *a* is a constant parameter. This means that the released energy (in joules) rises linearly with *d*.

As a negative aspect, non-zero remanent moment can lead to undesirable aggregation phenomena. Moreover, the use of nanospheres poses criticalities concerning the fulfilment of the biophysical constraint *H*_*a*_ *f* ≤ 5 · 10^9^ Am^−1^s^−1^. Due to the high irreversible fields, which result to be larger than 100 kA/m, only frequencies inadequate for hyperthermia applications could be applied. At the same time, if we reduce *H*_*a*_ below 100 kA/m, the reversal process becomes entirely reversible, without hysteresis effects and, consequently, with no energy release.

## Discussion

One of the issues of magnetic hyperthermia is the large quantity of magnetic nanomaterials required to release sufficient heat to the target area and then generate an appreciable temperature increment. Disk-shaped nanostructures can be a valid alternative to SPIONs, due the possibility of producing energy via hysteresis losses. However, their synthesis via nanolithography processes can be a very time-consuming and costly task, which needs to be addressed by proper design and optimization stages.

The study here reported, focused on permalloy nanostructures, demonstrates how micromagnetic simulations can be an efficient way to support fabrication steps in the engineering of optimized heating agents. First, micromagnetic modelling has enabled us to perform an extensive investigation of the influence of geometrical properties on the amount of heat generated via hysteresis losses. Second, it has provided useful information on remanence magnetization configuration and on its implications in aggregation phenomena. Third, it has allowed us to explore magnetization reversal process and determine saturation fields, which are required to obtain major hysteresis loops and thus maximize hysteresis losses. Regarding this last aspect, caution has been paid to not exceed acceptable biophysical limits for the maximum applicable field at a given frequency, focusing on the well-known constraint *H*_*a*_ *f* ≤ 5 · 10^9^ Am^−1^s^−1^.

With the aim of reaching a compromise between heat maximization and biophysical limit fulfilment, we have investigated permalloy nanostructures with different shapes (disk, cylinder and sphere) and dimensions, ranging up to some hundreds of nanometres. In all the analysed cases, the magnetization reversal takes place via the nucleation, motion and expulsion of a vortex.

Regarding disk-/cylinder-shaped nanostructures, optimal heating performances can be obtained with limited diameters and thicknesses, lower than 300 nm and 50 nm, respectively. Specific energy losses higher than 70 kJ/m^3^ have been predicted for nanodisks with 150 nm diameter and 30 nm thickness. This data corresponds to an SLP value of ∼400 W/g, obtainable under the exposure to a field with a frequency of 50 kHz and an amplitude of 100 kA/m.

The increase in diameter and thickness leads to a reduction in the specific energy losses. Moreover, for thicknesses around 100–150 nm, we have observed a strong increment of the vortex nucleation and expulsion fields, with a negative impact on the fulfilment of biophysical limits with frequencies adequate for hyperthermia applications. At remanence, all the analysed disk-/cylinder-shaped nanostructures are characterized by an out-of-plane vortex state with negligible magnetic moment and thus reduced possibility of aggregation.

Concerning nanospheres, high specific energy losses (∼65 kJ/m^3^) have been predicted for diameters around 100 nm, but in this case applied fields larger than 130 KA/m are required and a non-negligible magnetic moment appears. Moreover, the increase in nanosphere diameter leads to a strong reduction in the hysteresis losses, with very low contribution for sizes higher than 300 nm.

Finally, by means of a combined modelling and experimental analysis we have studied how magnetostatic interactions influence hysteresis losses, considering 30 nm thick nanodisks with diameters between 270 nm and 680 nm. For the nanodisks dispersed in ethanol solution, we have found that the nucleation and expulsion of vortexes are not synchronous, also due to the local misalignment with the applied field direction. Moreover, we have observed that the increment of volume concentration can be detrimental for specific energy losses, which halve when increasing the nanodisk concentration from 5% to 30%.

## Supplementary information

Supplementary Information for magnetic hyperthermia properties of permalloy nanostructures

## Data Availability

The datasets generated during and/or analysed during the current study are available from the corresponding author on reasonable request.
