# Supplementary material for: Influence of shape, size and magnetostatic interactions on the hyperthermia properties of permalloy nanostructures
Source: Sci Rep. 2019 Apr 29;9:6591. doi: 10.1038/s41598-019-43197-4 (PMC6488611; doi:10.1038/s41598-019-43197-4)
Supplement: Supplementary file 1 — Supplementary Information for magnetic hyperthermia properties of permalloy nanostructures [file 41598_2019_43197_MOESM1_ESM.docx]

***Supplementary Information***

Influence of shape, size and magnetostatic interactions on the hyperthermia properties of permalloy nanostructures

R. Ferrero^a,b^, A. Manzin*^a^, G. Barrera^a^, F. Celegato^a^, M. Coïsson^a^ and P. Tiberto^a^

^a^Istituto Nazionale di Ricerca Metrologica (INRIM), Strada delle Cacce 91, 10135, Torino, Italy.

^b^Politecnico di Torino, Corso Duca degli Abruzzi 24, 10129, Torino, Italy.

**Corresponding author address:* [*a.manzin@inrim.it*](mailto:a.manzin@inrim.it)*.*

*
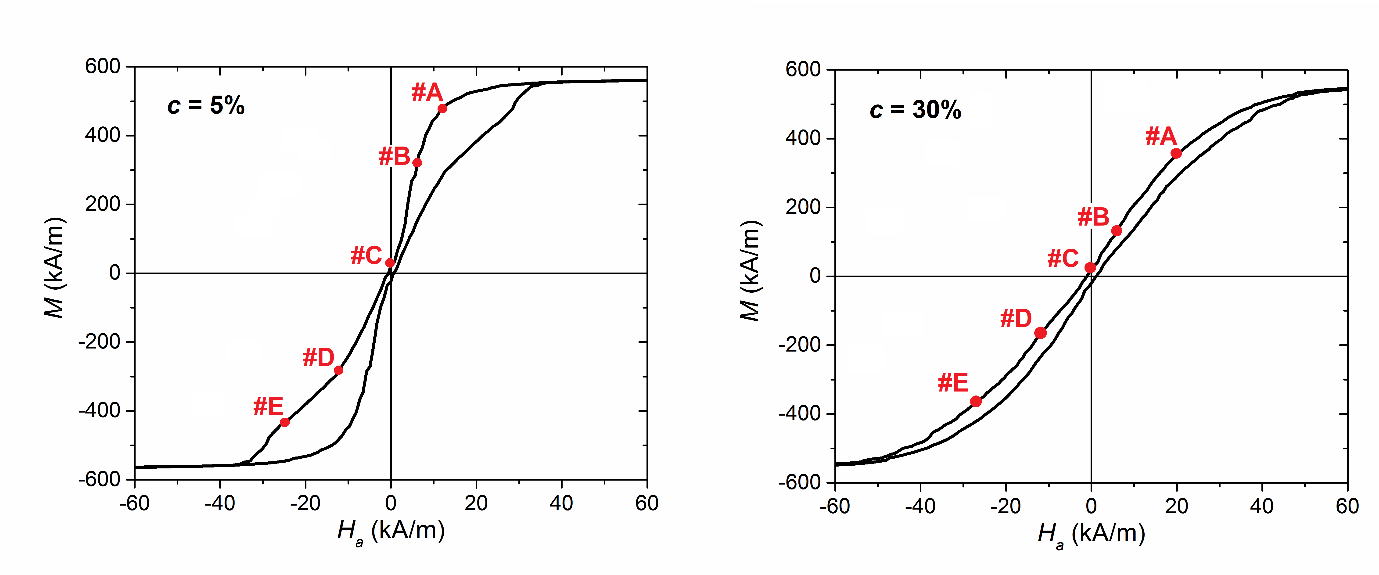
*

^
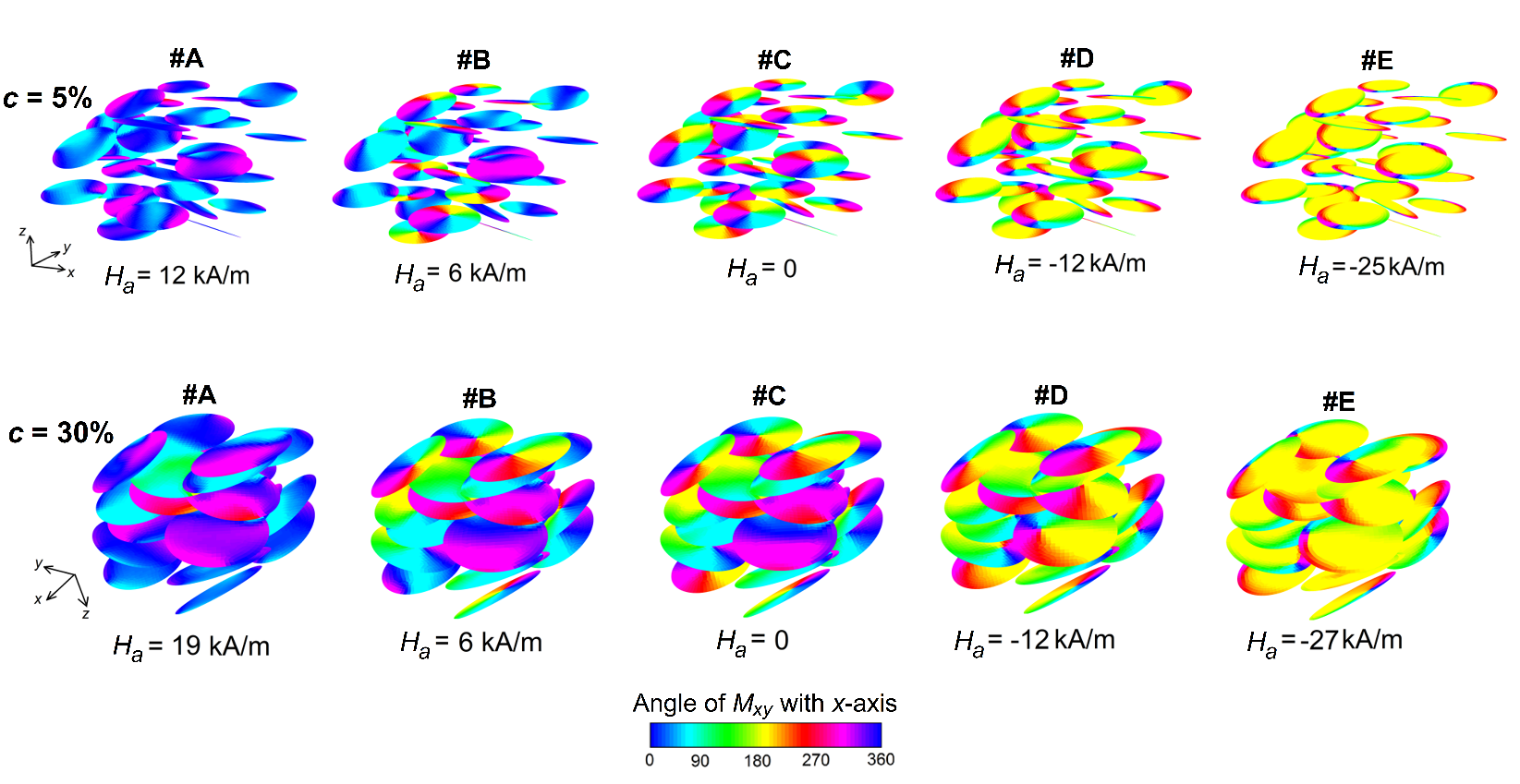
^

Fig. S1. Evolution of equilibrium states #A, #B, #C, #D and #E along the descending branch of the hysteresis loops of 680 nm diameter permalloy nanodisks randomly distributed in a 3D domain with two volume concentrations, namely 5% (top) and 30% (down). The hysteresis loops, reported on the top with the indication of the analysed equilibrium states, are calculated by applying the external field along the *x*-axis and by setting the temperature at 300 K. The colour bar represents the angle, in degrees, between magnetization component in the *xy*-plane (*M_xy_*) and *x*-axis.


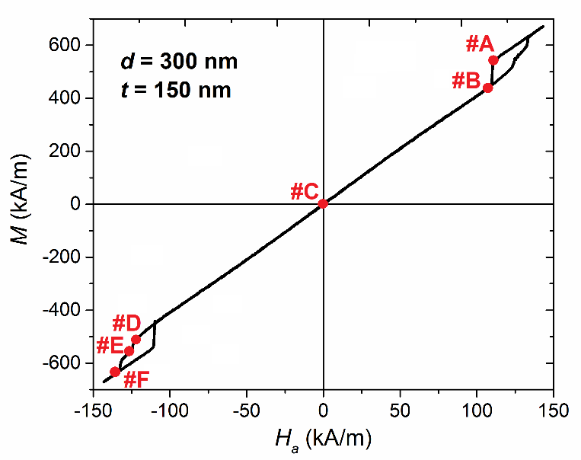


^
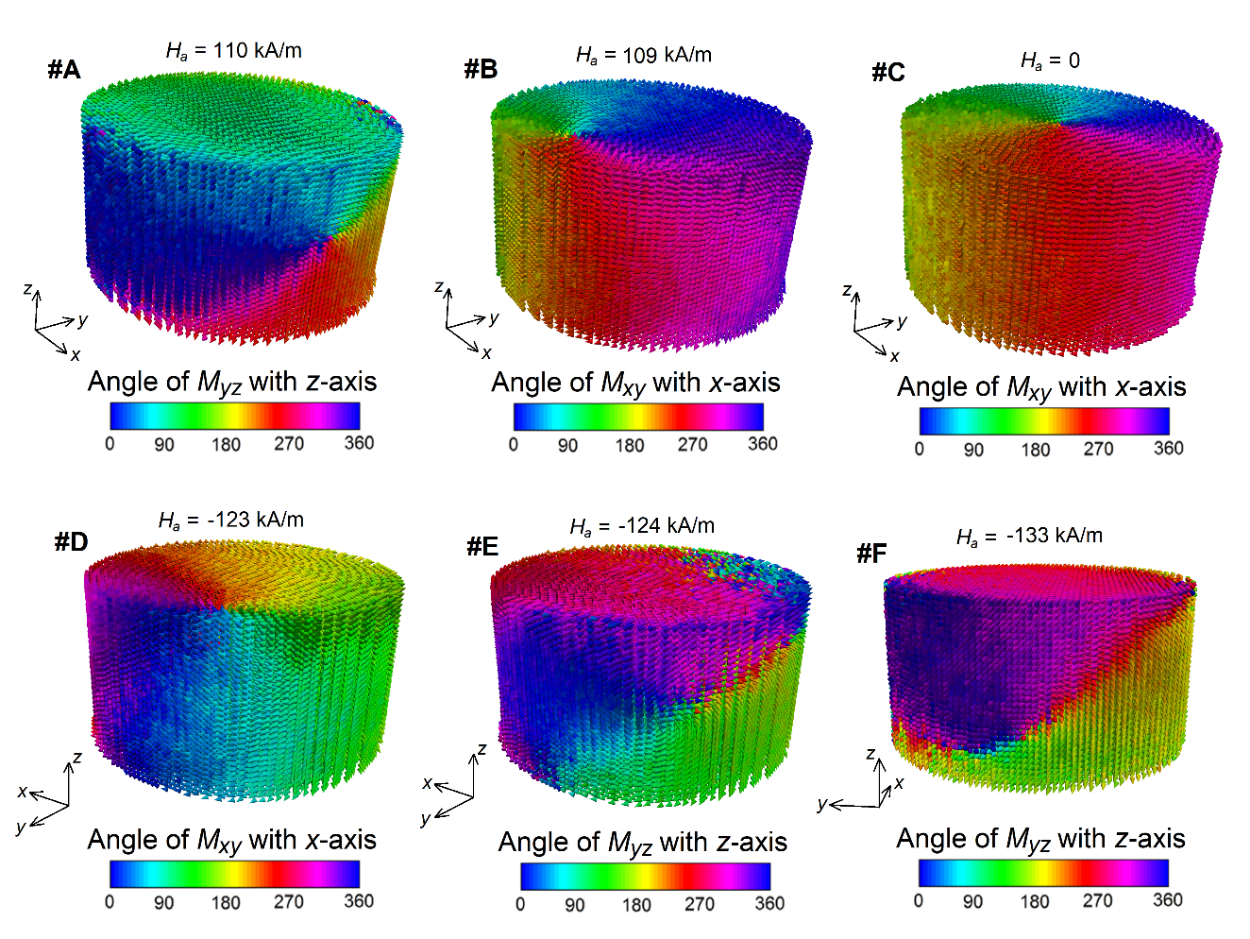
^

Fig. S2. Evolution of equilibrium states #A, #B, #C, #D, #E and #F along the descending branch of the hysteresis loop of a permalloy nanocylinder with a diameter of 300 nm and a thickness of
150 nm. The corresponding hysteresis loop, reported on the top with the indication of the analysed equilibrium states, is calculated by applying the external field along the *x*-axis and by setting the temperature at 300 K. The colour bars represent the angle, in degrees, between the indicated
in-plane magnetization components and axes. Depending on the equilibrium state, the 3D view is varied to better illustrate the magnetization spatial distribution.

As described in the manuscript, the magnetization reversal starts with the formation of a third-order buckle state, which evolves into an in-plane vortex (#A). At the first irreversible jump, the in-plane vortex transforms into a vortex with out-of-plane core (#B). Along the reversible part of the loop, this out-of-plane vortex moves orthogonally to the applied field up to the opposite side (#D), where it is expelled, evolving into a third-order buckle state (#E). The final irreversible jump corresponds to the shift of the buckle state towards the direction of the applied field (#F).


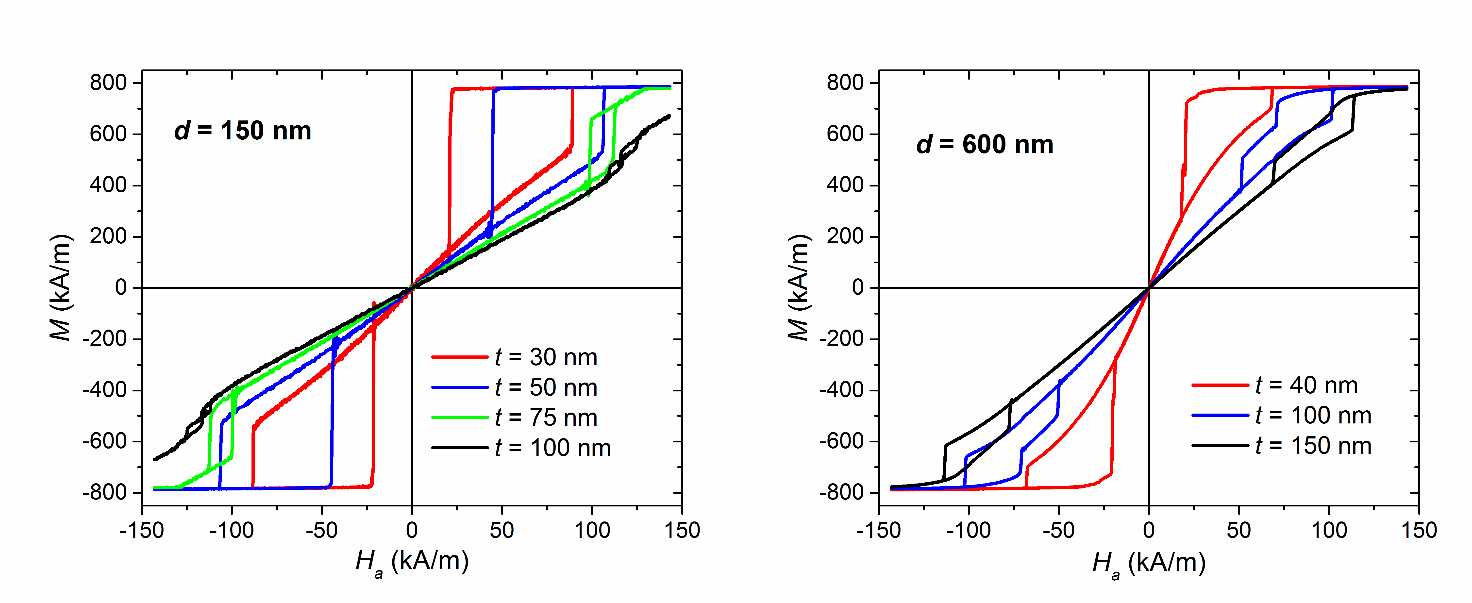


Fig. S3. Comparison of hysteresis loops calculated as a function of thickness *t* for nanocylinders with diameter *d* equal to 150 nm (left) and 300 nm (right). The simulations are performed by setting the temperature at 300 K.


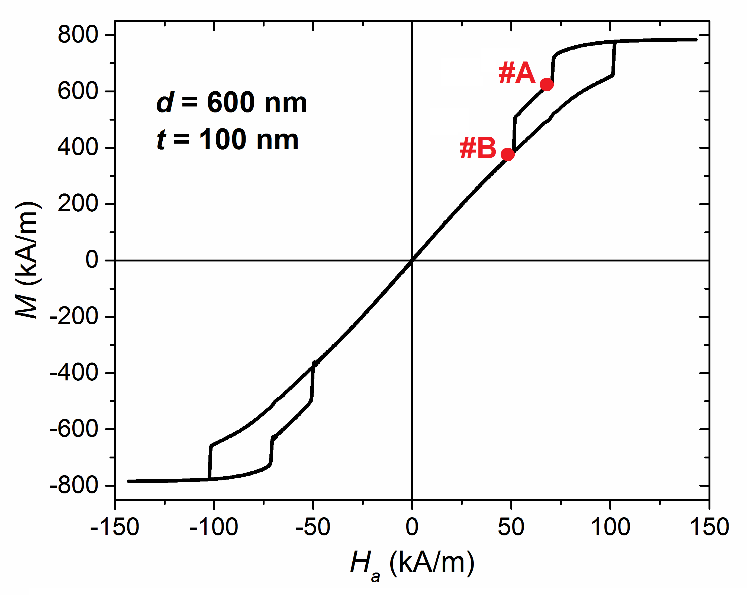


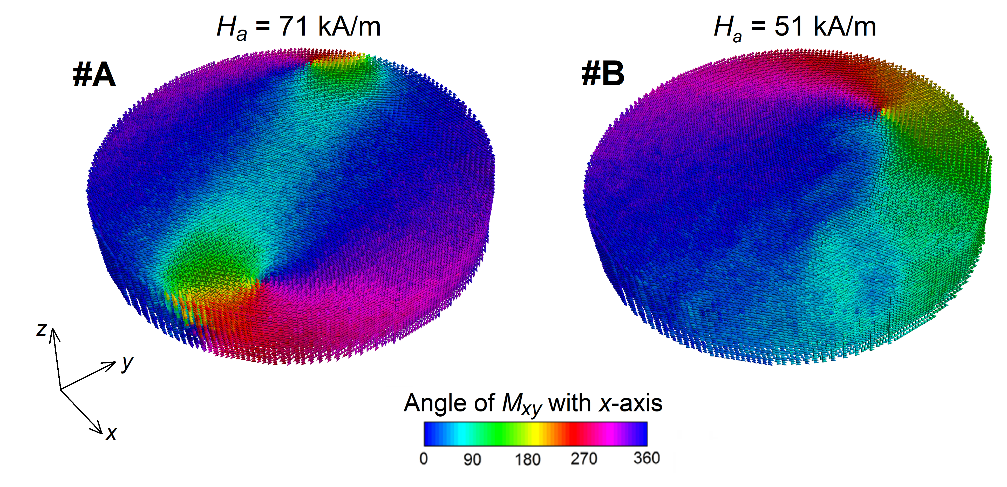


Fig. S4. Magnetization configurations at the equilibrium states immediately after the first irreversible jump (left) and the second one (right) for a permalloy nanocylinder with a diameter of 600 nm and a thickness of 100 nm. The corresponding hysteresis loop is reported on the top with the indication of the analysed equilibrium states. The colour bar represents the angle, in degrees, between magnetization component in the *xy*-plane (*M_xy_*) and *x*-axis.


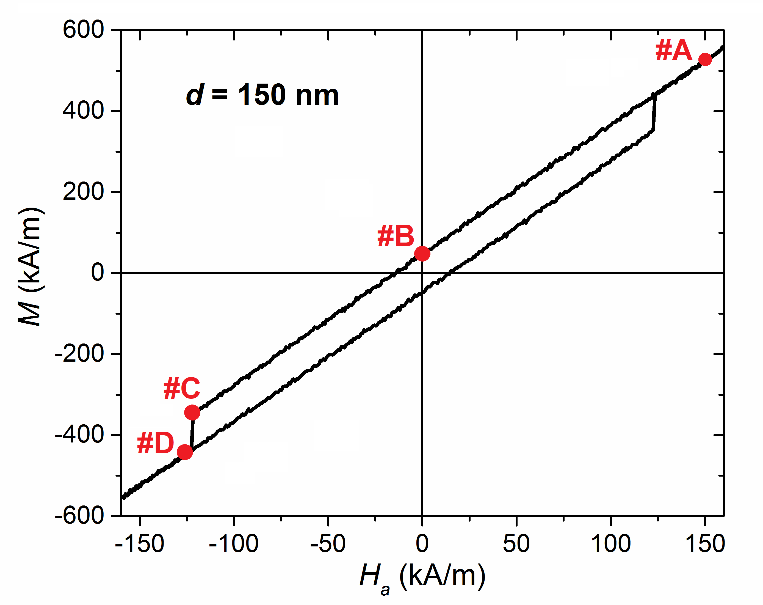

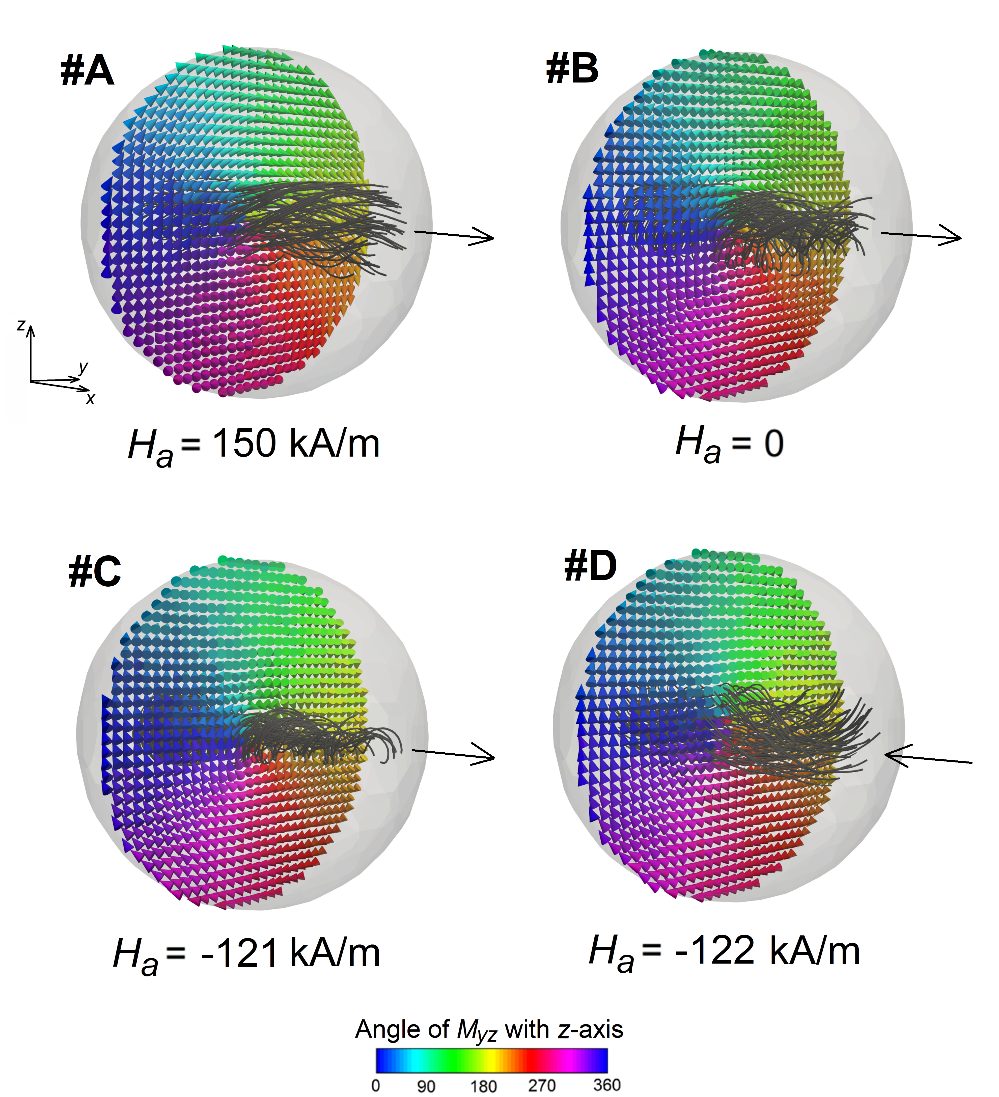


Fig. S5. Evolution of equilibrium states #A, #B, #C and #D along the descending branch of the hysteresis loop of a 150 nm sized permalloy nanosphere. The hysteresis loop is calculated by applying the external field along the *x*-axis and by setting the temperature at 300 K. Magnetization vector distribution is reported for the central *yz*-plane. The colour bar represents the angle, in degrees, between magnetization component in the *yz*-plane (*M_yz_*) and *z*-axis. The streamlines represent the vortex core, whose magnetization orientation is described by the arrow. The magnetic configurations on the bottom refer to the equilibrium states before (left) and after (right) the irreversible jump, which leads to the inversion of the vortex core magnetization.
